# Supplementary material for: Facile Synthesis of 3-(Azol-1-yl)-1-adamantanecarboxylic Acids—New Bifunctional Angle-Shaped Building Blocks for Coordination Polymers
Source: Molecules. 2019 Jul 26;24(15):2717. doi: 10.3390/molecules24152717 (PMC6695720; doi:10.3390/molecules24152717)
Supplement: Supplementary file 1 [file molecules-24-02717-s001.pdf]

Supplementary materials

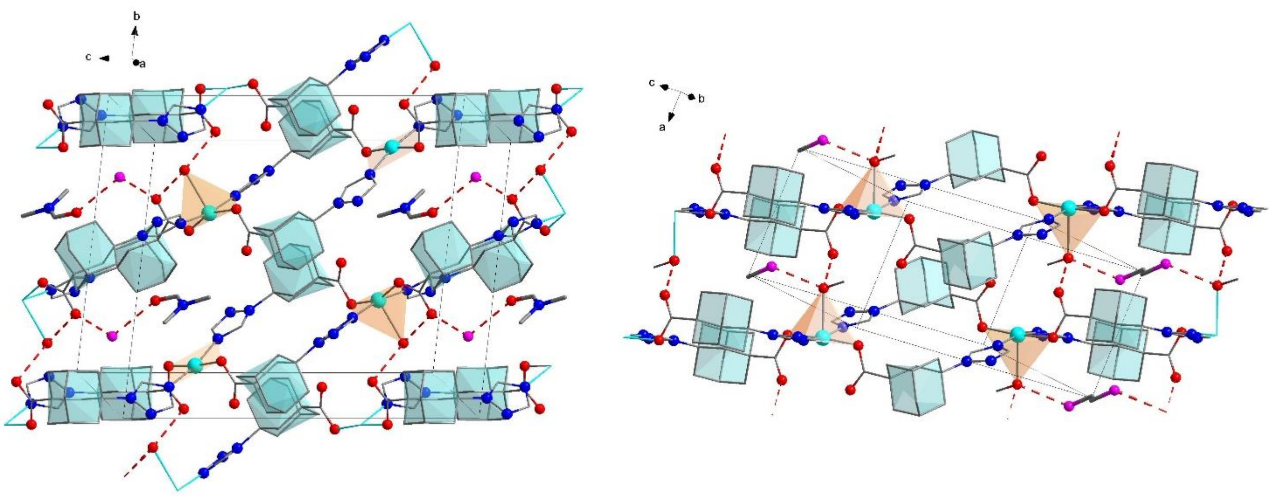

a) b)

Fig S1. Representation of a layer built from 1D chains of the complexes an solvate molecules for **1** (left) and **2** (right). Oxygen atoms of solvate molecules are colored pink, dashed red lines indicate hydrogen bonds. Adamantane units are colored blue, coordination polyhedra of the central atoms are colored orange. Hydrogen atoms have been omitted for clarity.

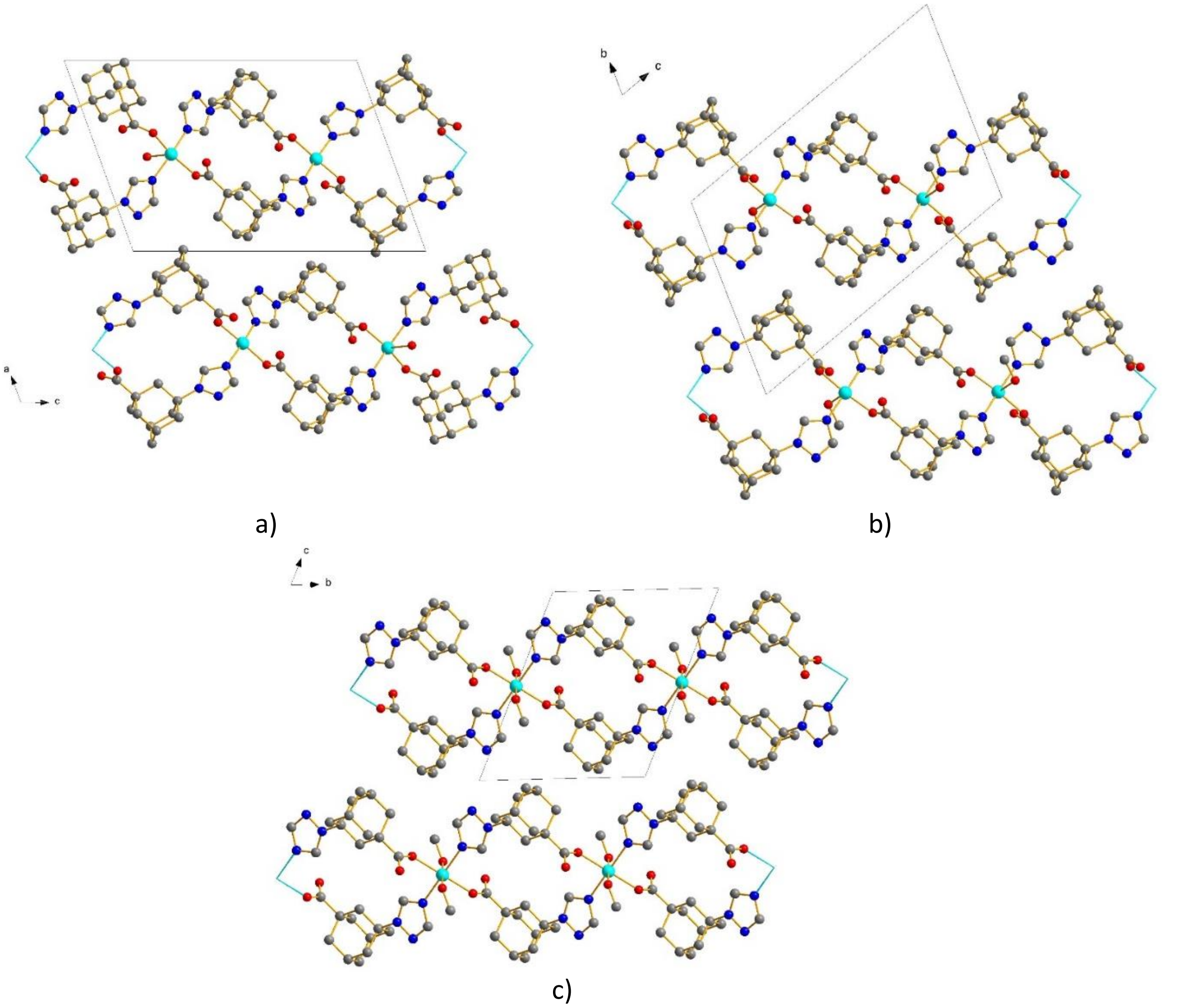

a) b)

c)

Fig. S2. Representation of 1D chains of **1** (a), **2** (b) and **3** (c) showing their similarity. Hydrogen atoms and solvate molecules are omitted

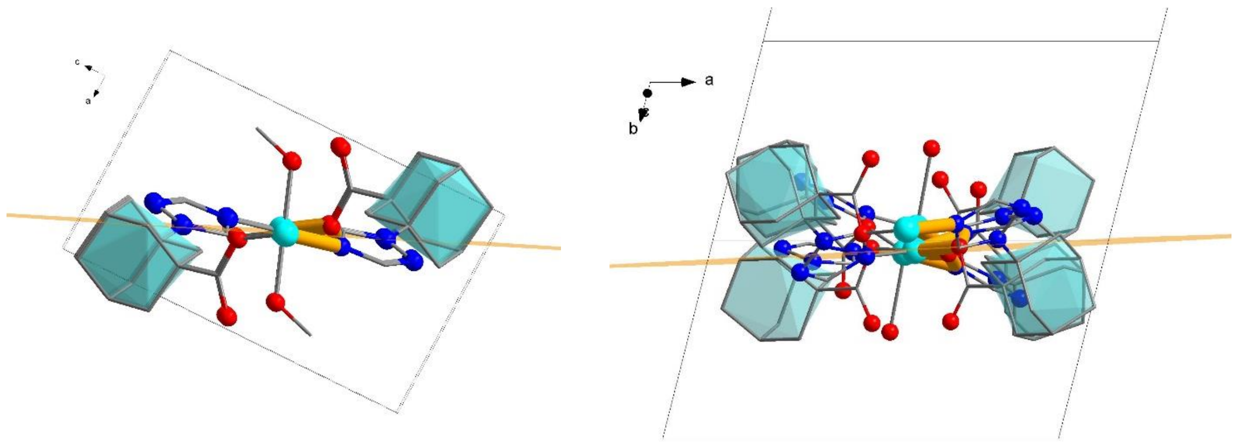

a) b)

Fig. S3. Structure view along 1D chain showing difference between **3** (a) and **1**–**2** (b, on the example of **1**). CuNO triangles and their mean planes marked orange. Adamantane units are coloured blue, hydrogen atoms and solvate molecules are omitted.

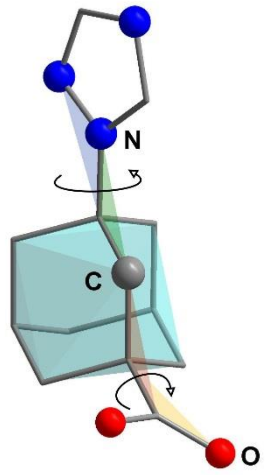

Fig. S4. Representation of torsion angles of **trzadc**$^-$ and **mtrzadc**$^-$ ligands on the example of **trzadc**$^-$.

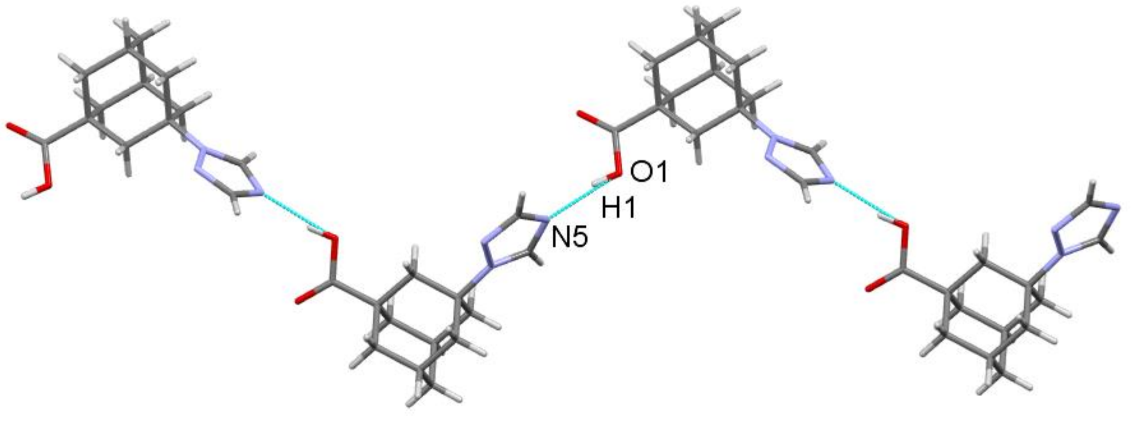

Fig. S5. Intermolecular hydrogen bonds between molecules of **trzadcH**.

Table S1. Torsion angles (deg.) for **trzadc**$^-$ and **mtrzadc**$^-$ ligands in the complexes **1**–**4**.

	N–N–C–C	O–C–C–C
**1**	136.4, 162.7, 175.1, 174.4	68.3, 67.0, 72.7, 65.7
**2**	169.4, 179.8	66.2, 86.3
**3**	165.4	74.4
**4**	70.0, 172.7, 49.5	87.1, 83.8, 48.0

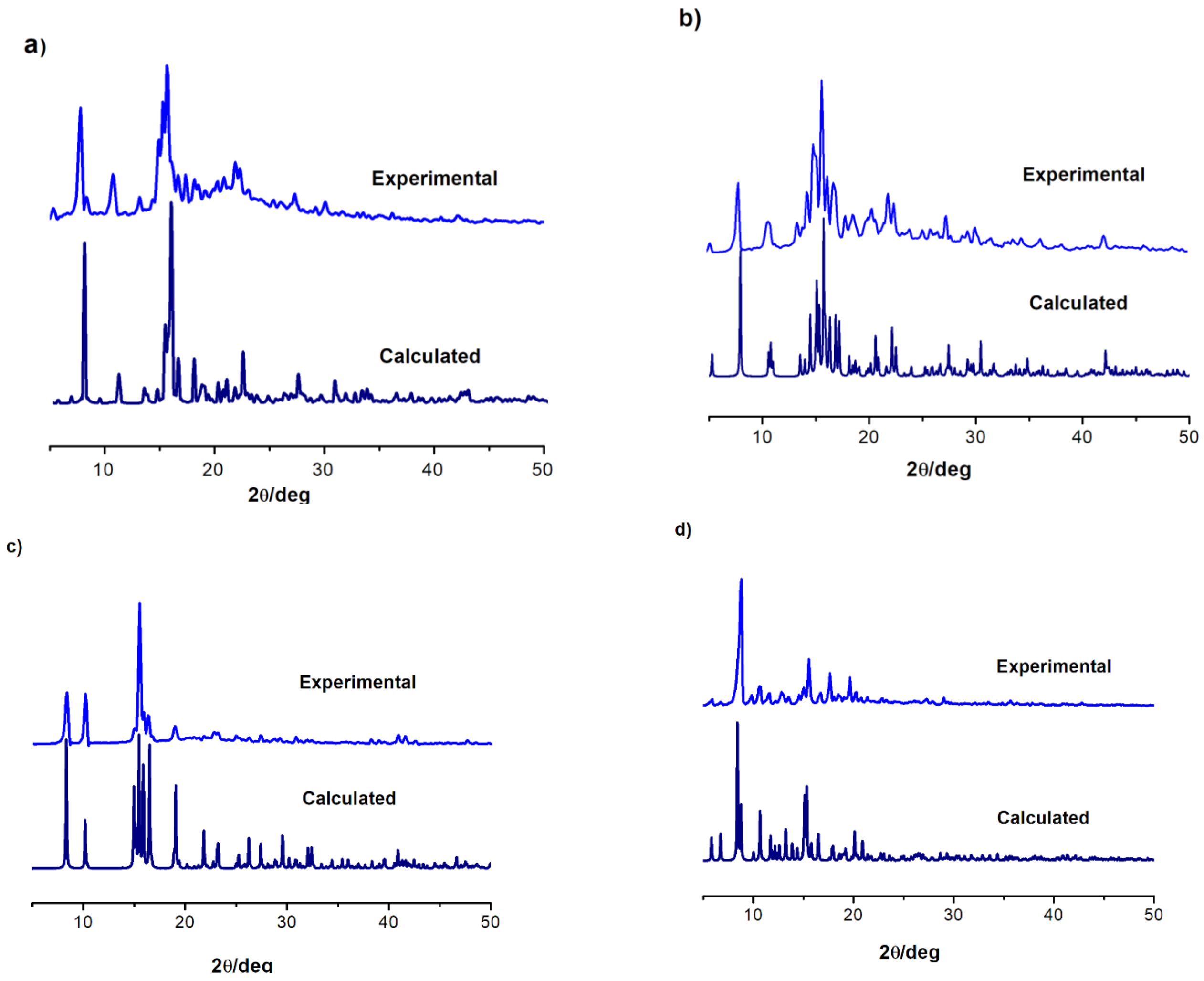

Fig. S6 The PXRD patterns for compounds **1-4** (a-d)

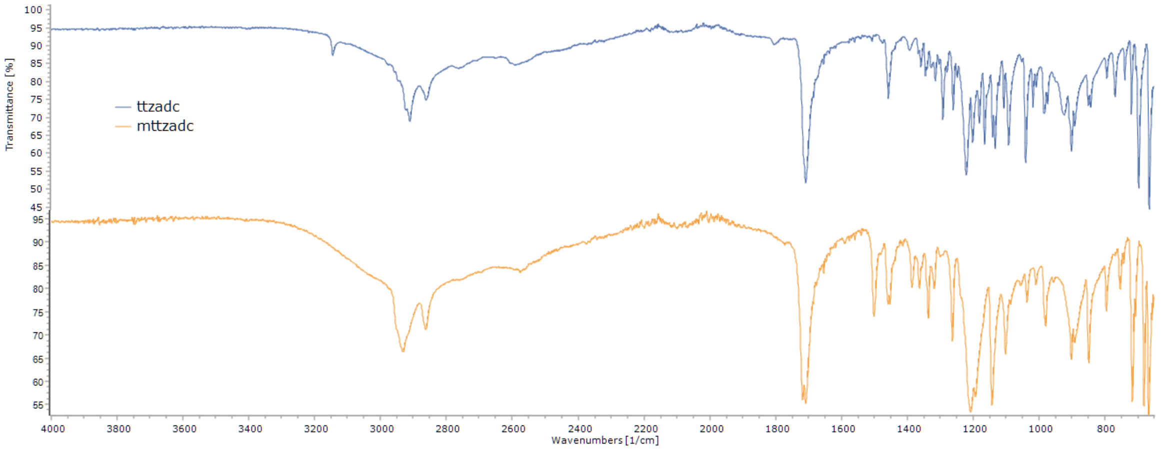

a)

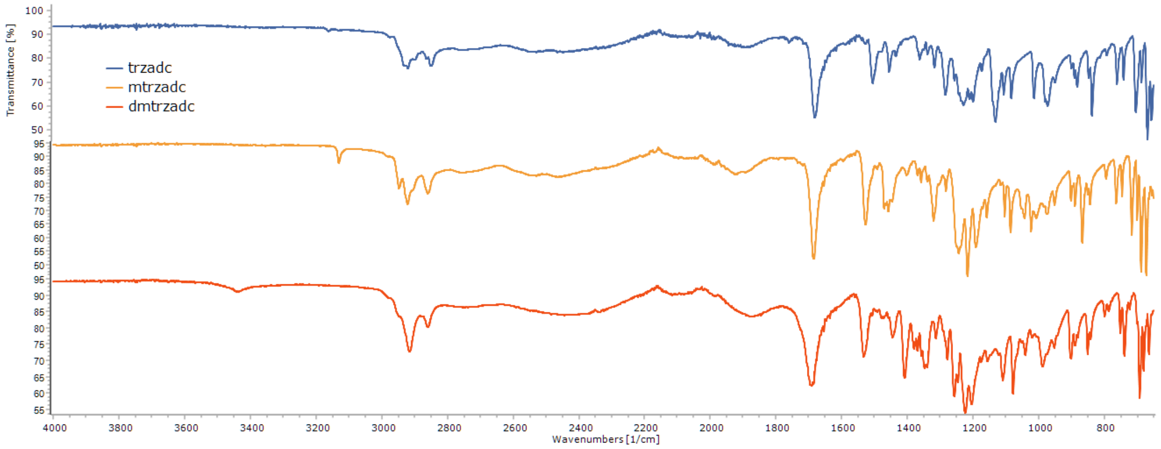

b)

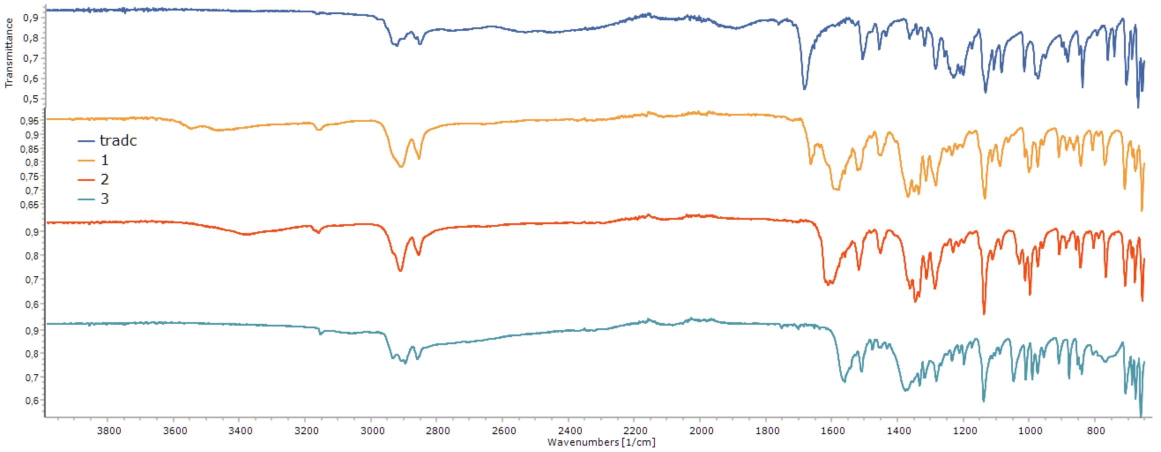

c)

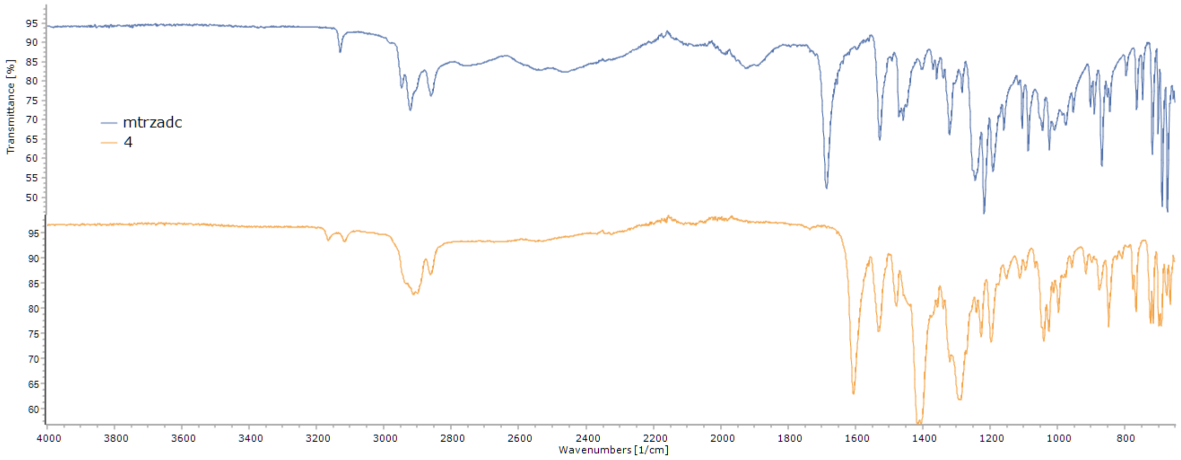

d)

Fig S7 IR Spectra of **trzadc**, **mtrzadc**, **dmtradc** (a); IR spectra of **ttzadc**, **mttzadc** (b); IR spectra of trzadc and compounds **1-3** (c); IR spectra of **mtrzadc** and compound **4** (d).

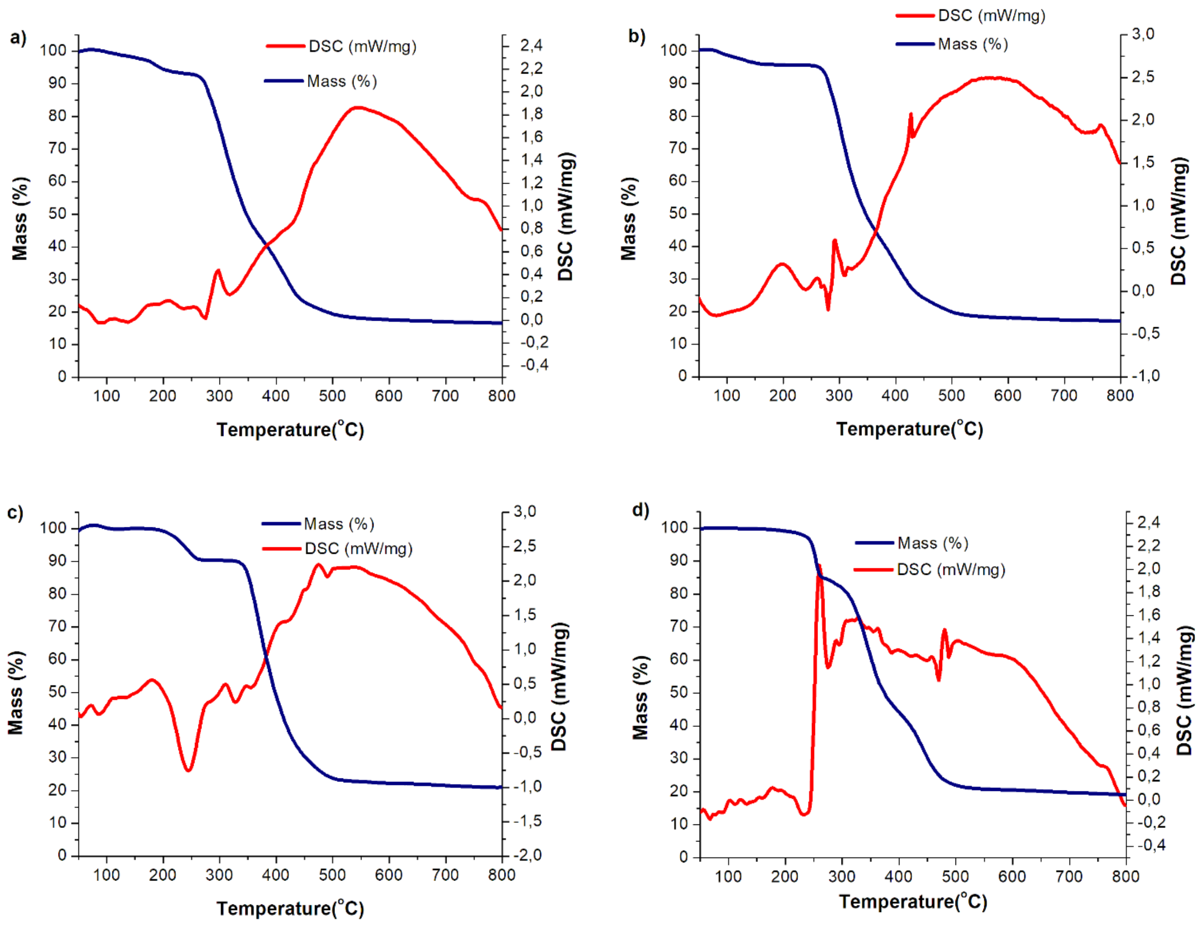

Fig S8. TGA/DSC curves for compounds **1-4** (a-d).

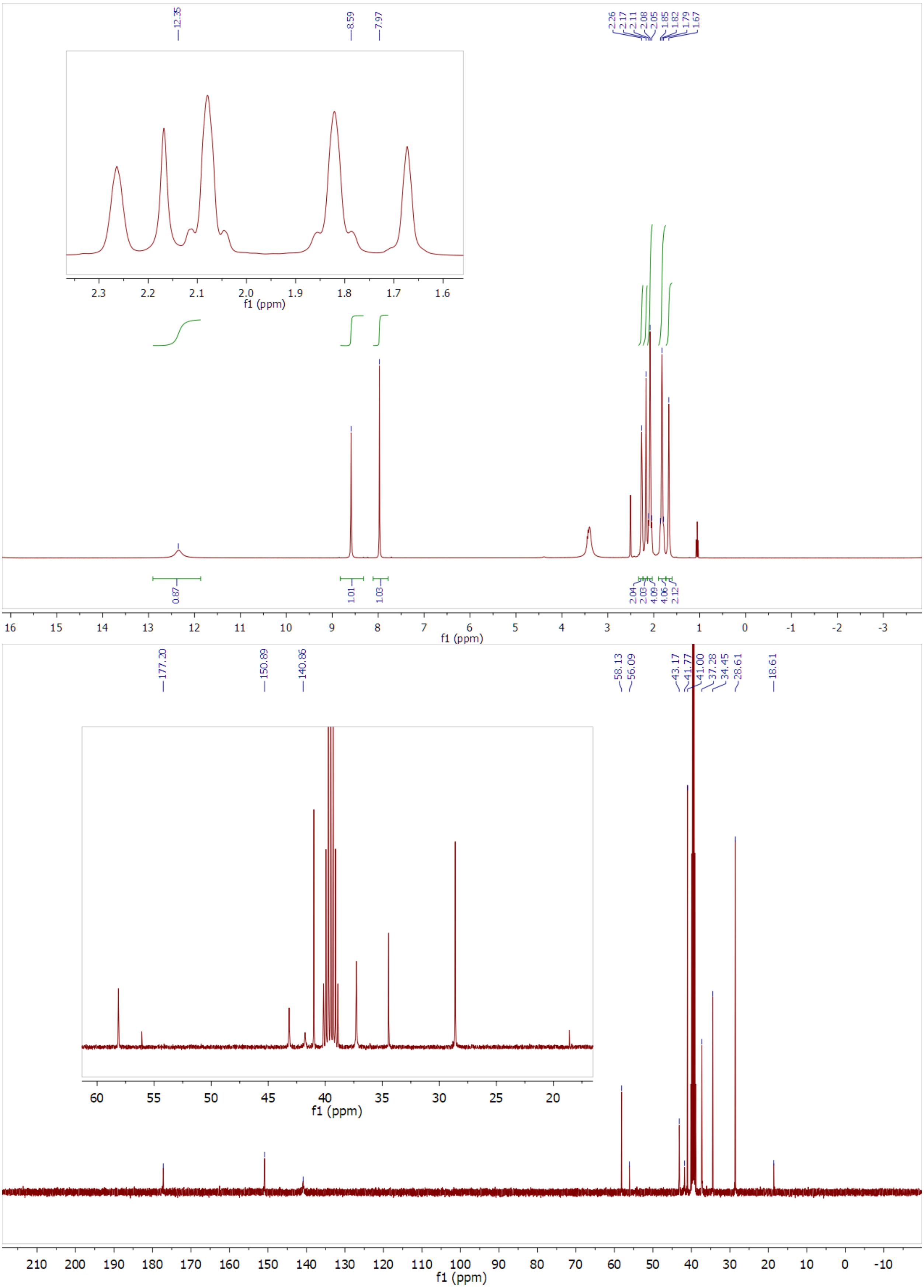

Fig. S9 NMR Spectra of **trzadc**

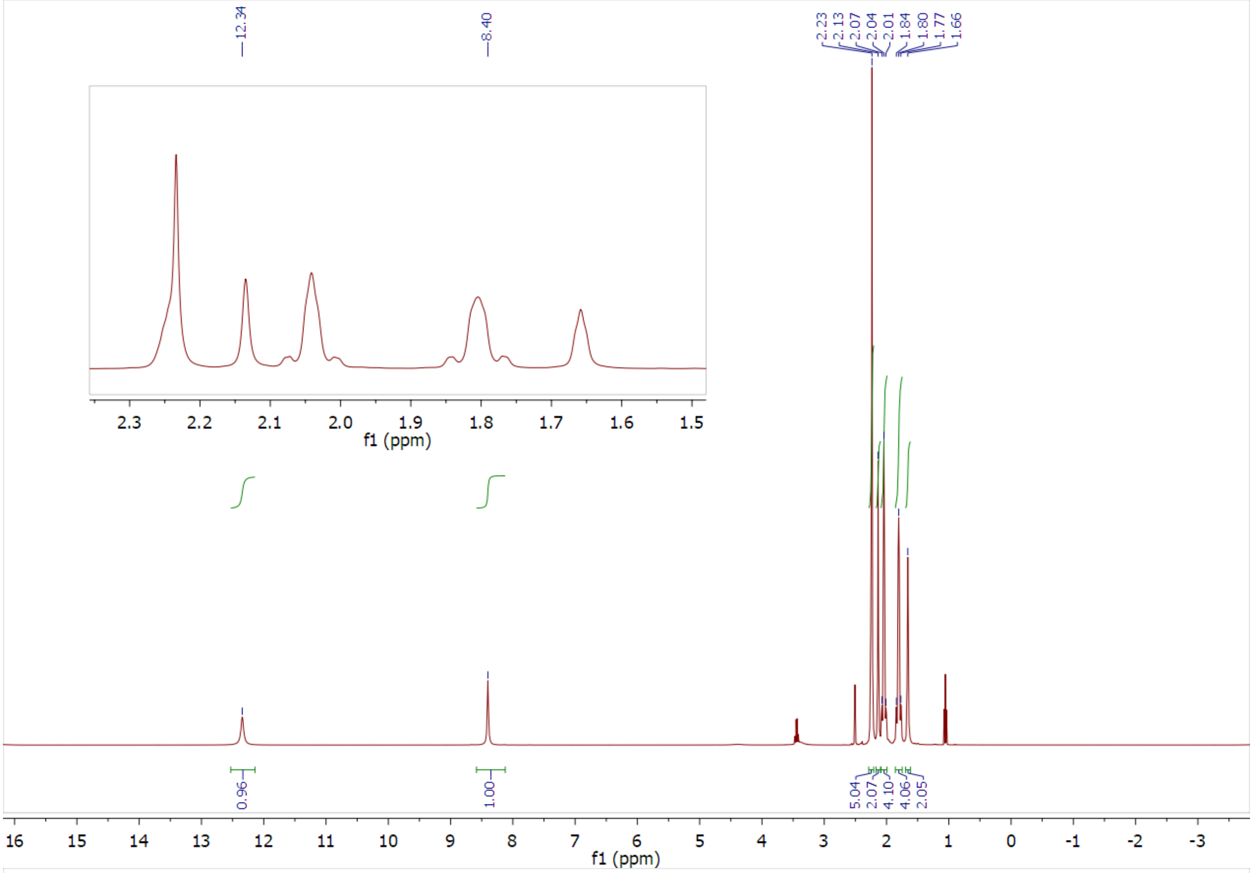

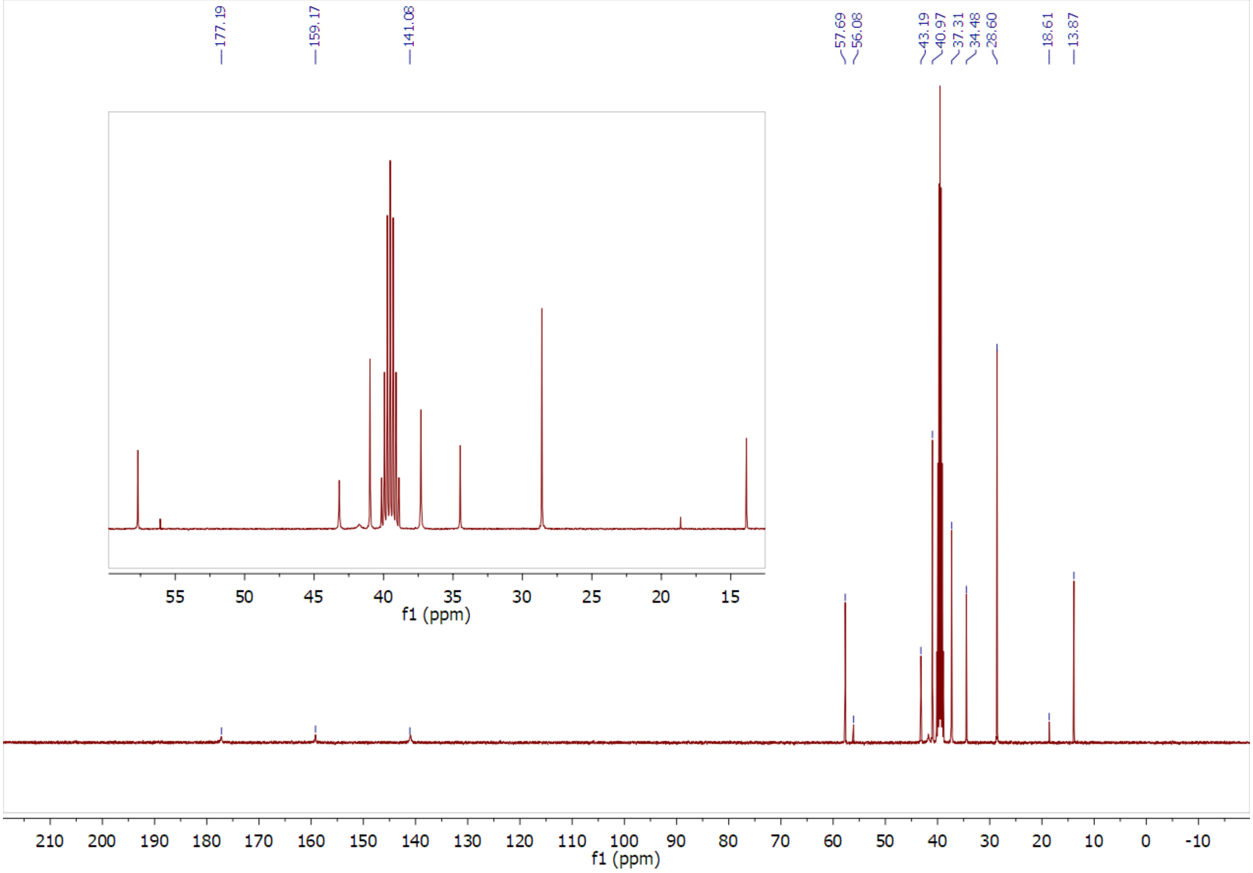

Fig. S10 NMR Spectra of **mtrzadc**

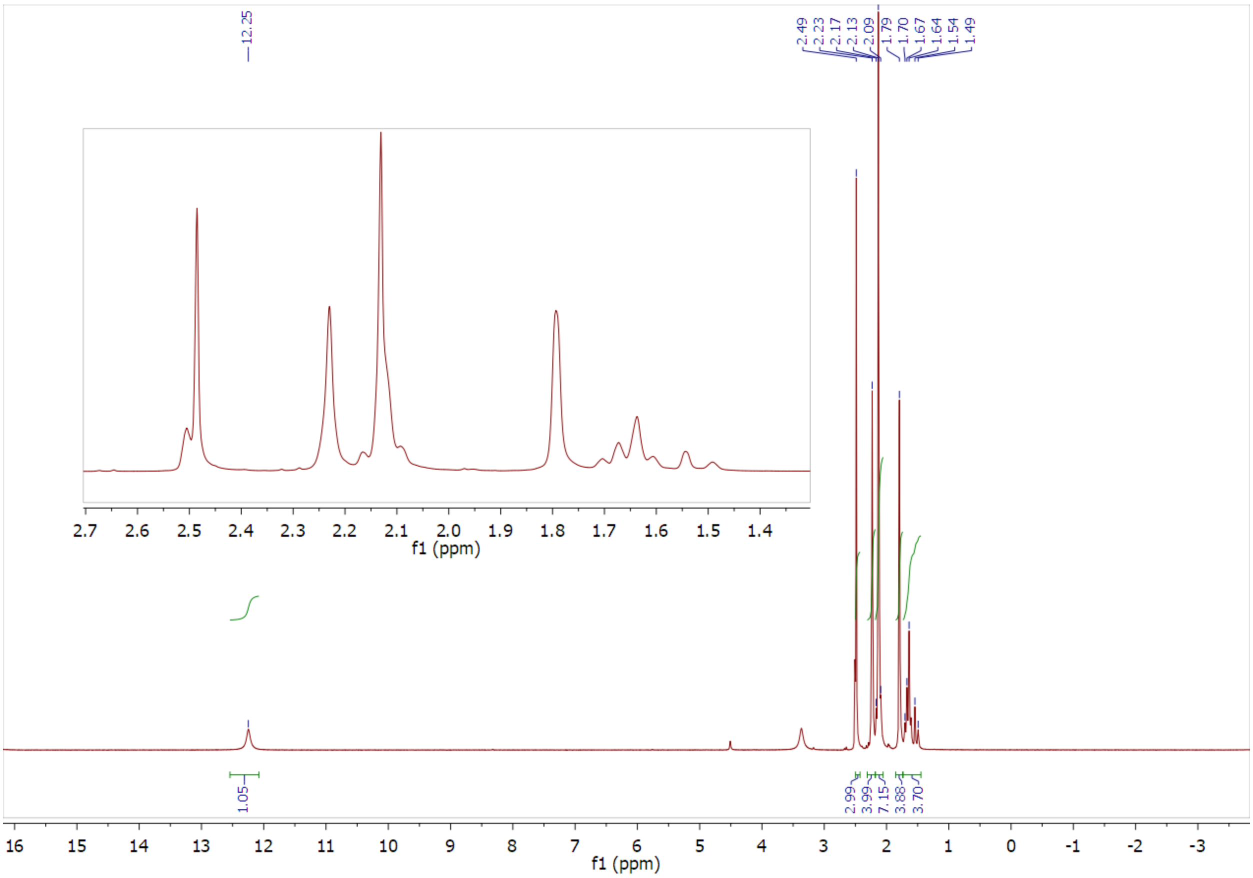

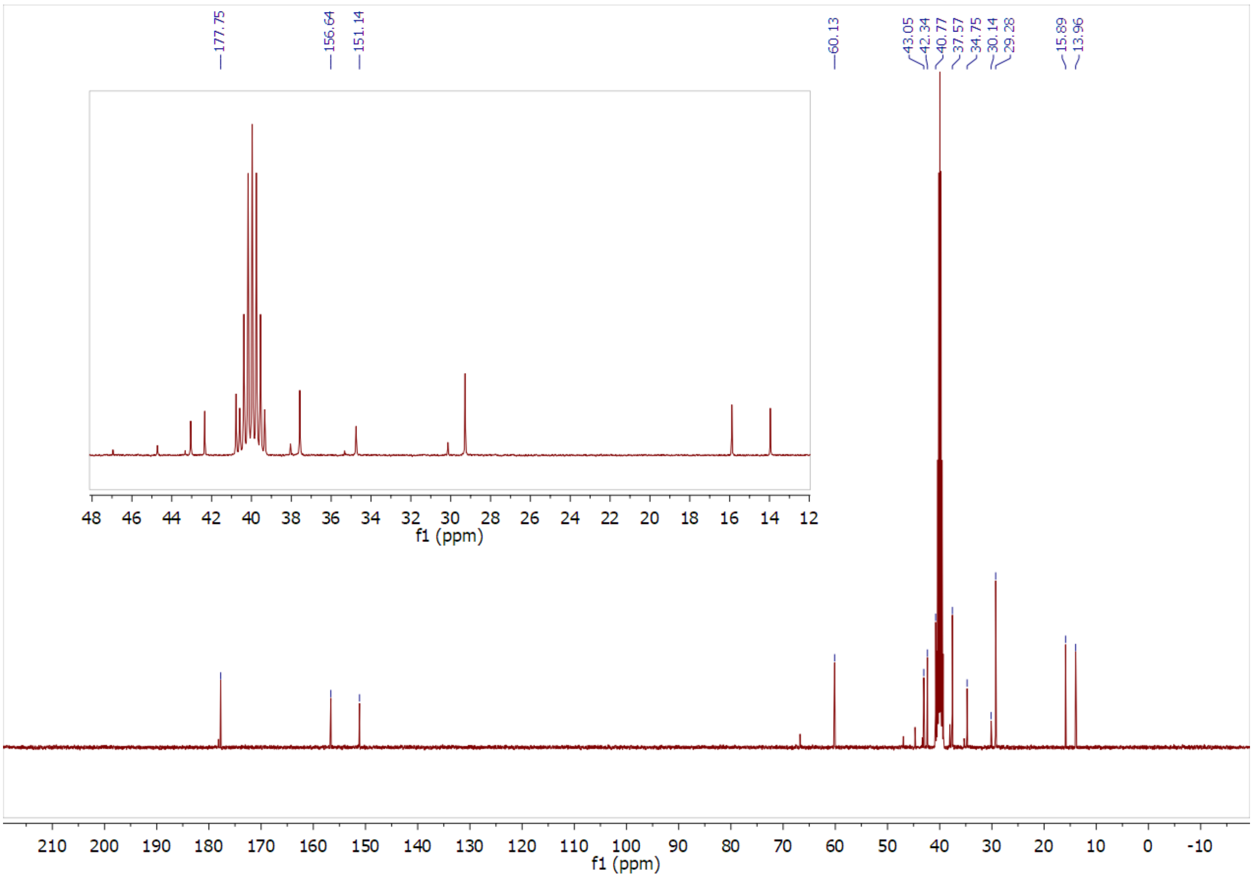

Fig. S11 NMR Spectra of **dmtradc**

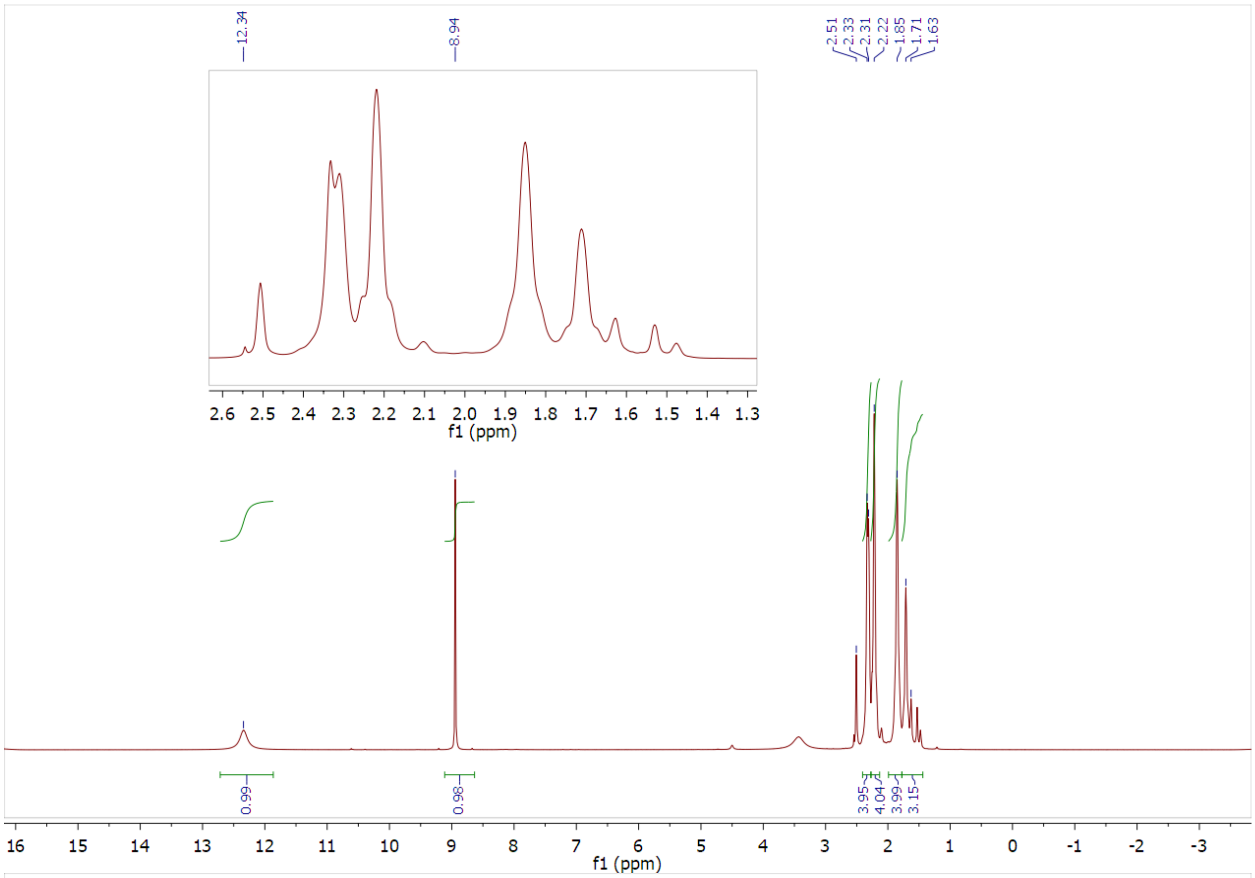

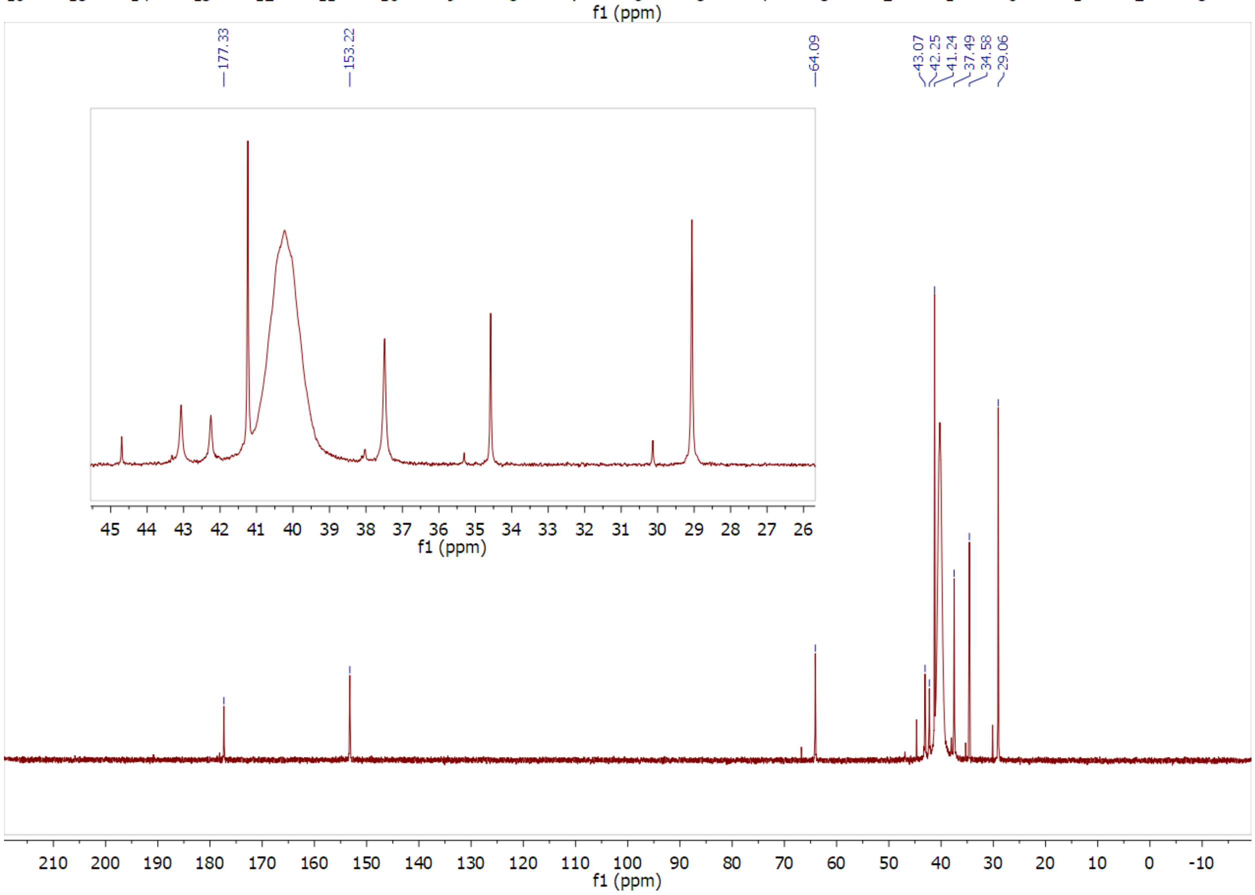

Fig. S12 NMR spectra of **ttzadc**

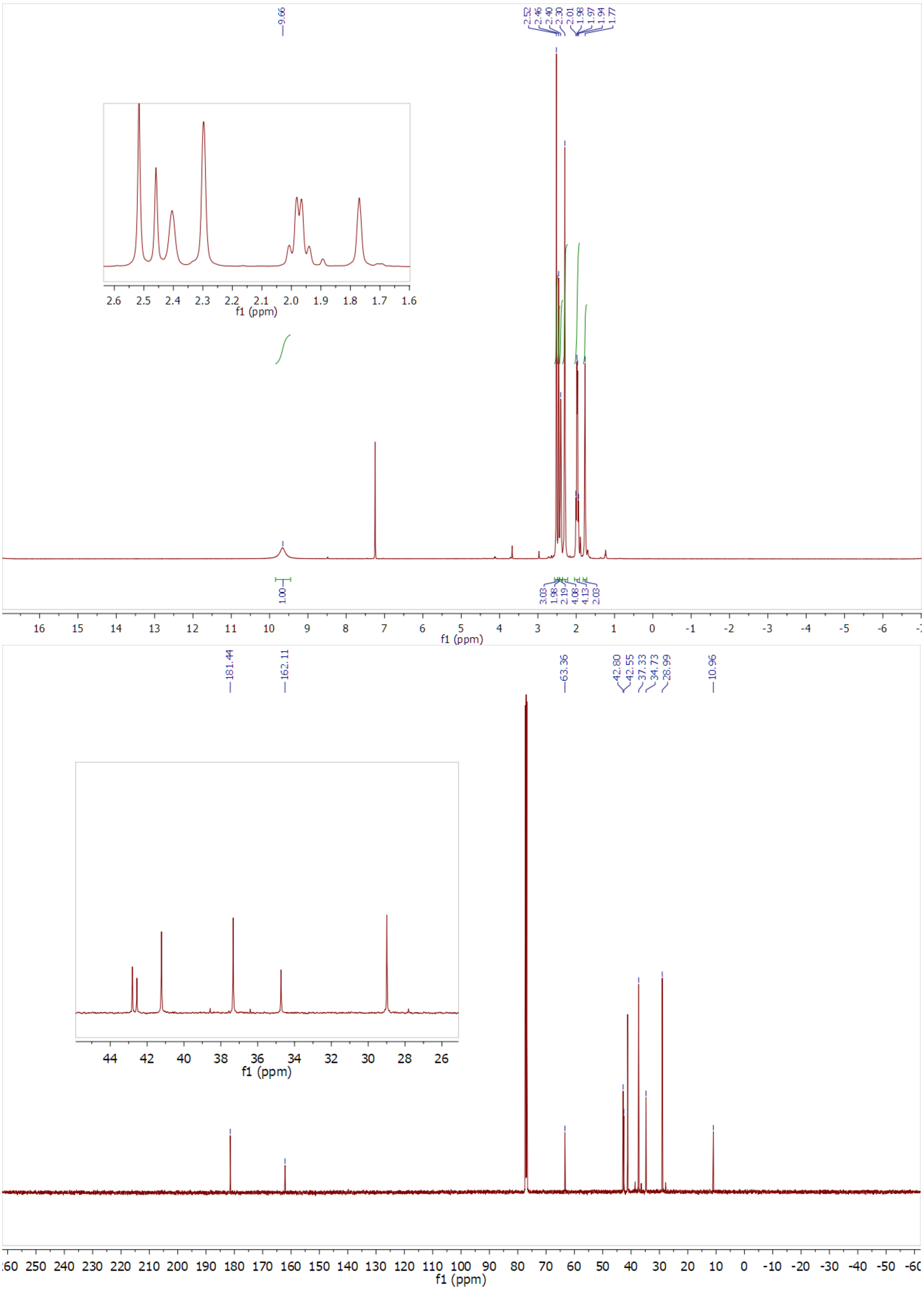

Fig. S13 NMR Spectra of **mttzadc**

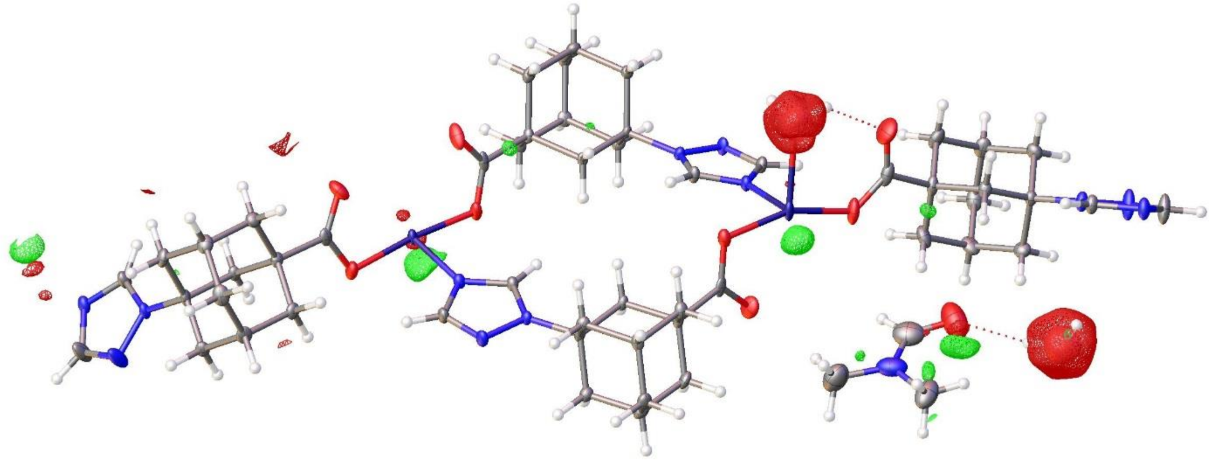

Fig. S14. Residual electron density map (0.55 eÅ^{-3} level) for the complex **1** with full occupancy of coordinated and solvate water molecules. Green and red area show excess or lack of electron density, respectively.
